# Highlight report: Import of fatty acids by metastasizing tumor cells

**DOI:** 10.17179/excli2018-1870

**Published:** 2018-11-19

**Authors:** Tim Brecklinghaus

**Affiliations:** 1 Leibniz Research Centre for Working Environment and Human Factors at the Technical University of Dortmund (IfADo), 44139, Dortmund, Germany

## ⁯⁯

Recently Pascual and colleagues have contributed a study about the identity of cells that initiate metastasis (Pascual et al., 2017[22]). They identified a cell type in human oral carcinomas with the following properties: (1) slow-cycling, (2) CD44-bright, (3) low expression of mesenchymal genes, (4) ability to initiate metastasis in mouse models and (5) high expression of the fatty acid receptor CD36. CD36 is a membrane protein on the surface of many mammalian cells that imports fatty acids (Yang et al., 2018[34]; Umbarawan et al., 2018[31]; Son et al., 2018[28]). CD36 has been shown to be critical for supply with fatty acids and for maintenance of energy metabolism under numerous conditions (Wen et al, 2017[33]; Chen et al, 2016[5]; Nakatani et al., 2015[21]; le Foll et al., 2015[15], 2013[14]). In their recent study Pascual et al. report that neutralizing antibodies against CD36 reduce the formation of metastasis in orthotopic mouse models of oral cancer (Pascual et al., 2017[22]). A further finding of this study is that NOD scid gamma mice developed larger lymph node metastases in a CD36 dependent manner, when the mice received a high-fat diet. Moreover, the authors report that CD36 positive cells are metastasis initiating and are characterized by a lipid metabolism signature (Pascual et al., 2017[22]). Based on publicly available data, high expression of CD36 was associated with poor disease-free survival in breast, lung and urinary bladder cancer (Pascual et al., 2017[22]).

In the past decade much progress has been made in understanding the principles that control formation of metastases (McGranahan et al, 2017[20]; Lambert et al., 2017[13]; Adawy, 2017[1]; Marchan, 2012[18]; Cadenas, 2012[2]; Mantovani et al., 2017[17]; Zhan et al, 2017[35]). It is generally accepted that the cellular and humoral immune system play an important role in preventing metastasis (Schmidt et al., 2018[25], 2012[24], 2008[23]; Godoy et al., 2014[6]; Heimes et a., 2017[8][9]; Sicking et al., 2014[26]). 

In many tumor types high expression of proliferation associated genes has been shown to lead to an increased risk of metastasis (Schmidt et al., 2008[23]; Siggelkow et al., 2012[27]; Jabs et al., 2017[11]; Wei et al,, 2017[32]; Knaack, et al, 2018[12]). Moreover, high expression of antioxidative factors (Cadenas et al, 2010[3]), disturbed expression of genes involved in the control of circadian rhythm (Cadenas et al., 2014[4]) and actin binding proteins (Stock et al., 2015[30]) are associated with shorter metastasis-free interval.Different principles have been shown to control breast cancer metastasis that occurs within the first three years after the primary tumor or later (Hellwig et al., 2016[10]; Hammad et al., 2016[7]). Finally, factors involved in glycerophospholipid metabolism have been shown to influence the capacity of tumor cells to migrate, attach to surfaces and to metastasize (Stewart et al., 2012[29]; Lesjak et al., 2014[16]; Marchan et al., 2017[19]).

In conclusion, Pascual and colleagues bring forward an interesting concept that metastasis-initiating tumor cells rely on dietary lipids in a CD36 dependent manner. It remains to be shown, whether CD36 is a promising target for anti-cancer therapy. 
